# YPED: An Integrated Bioinformatics Suite and Database for Mass Spectrometry-based Proteomics Research

**DOI:** 10.1016/j.gpb.2014.11.002

**Published:** 2015-02-21

**Authors:** Christopher M. Colangelo, Mark Shifman, Kei-Hoi Cheung, Kathryn L. Stone, Nicholas J. Carriero, Erol E. Gulcicek, TuKiet T. Lam, Terence Wu, Robert D. Bjornson, Can Bruce, Angus C. Nairn, Jesse Rinehart, Perry L. Miller, Kenneth R. Williams

**Affiliations:** 1 W.M. Keck Foundation Biotechnology Resource Laboratory, School of Medicine, Yale University, New Haven, CT 06510, USA; 2 Department of Molecular Biophysics & Biochemistry, Yale University, New Haven, CT 06520, USA; 3 Yale Center for Medical Informatics, School of Medicine, Yale University, New Haven, CT 06510, USA; 4 Department of Anesthesiology, School of Medicine, Yale University, New Haven, CT 06510, USA; 5 Department of Emergency Medicine, School of Medicine, Yale University, New Haven, CT 06510, USA; 6 VA Connecticut Healthcare System, West Haven, CT 06516, USA; 7 Department of Computer Science, Yale University, New Haven, CT 06520, USA; 8 Yale Center for Genome Analysis, West Campus, Yale University, Orange, CT 06477, USA; 9 Yale West Campus Analytical Core, West Campus, Yale University, West Haven, CT 06516, USA; 10 Yale Bioinformatics Resource, School of Medicine, Yale University, New Haven, CT 06510, USA; 11 Department of Psychiatry, School of Medicine, Yale University, New Haven, CT 06510, USA; 12 Department of Cellular & Molecular Physiology, School of Medicine, Yale University, New Haven, CT 06510, USA; 13 Systems Biology Institute, Yale University, West Haven, CT 06516, USA

**Keywords:** Proteomics, Database, Bioinformatics, Mass spectrometry, Repository, Spectral library

## Abstract

We report a significantly-enhanced bioinformatics suite and database for proteomics research called Yale Protein Expression Database (YPED) that is used by investigators at more than 300 institutions worldwide. YPED meets the data management, archival, and analysis needs of a high-throughput mass spectrometry-based proteomics research ranging from a single laboratory, group of laboratories within and beyond an institution, to the entire proteomics community. The current version is a significant improvement over the first version in that it contains new modules for liquid chromatography–tandem mass spectrometry (LC–MS/MS) database search results, label and label-free quantitative proteomic analysis, and several scoring outputs for phosphopeptide site localization. In addition, we have added both peptide and protein comparative analysis tools to enable pairwise analysis of distinct peptides/proteins in each sample and of overlapping peptides/proteins between all samples in multiple datasets. We have also implemented a targeted proteomics module for automated multiple reaction monitoring (MRM)/selective reaction monitoring (SRM) assay development. We have linked YPED’s database search results and both label-based and label-free fold-change analysis to the Skyline Panorama repository for online spectra visualization. In addition, we have built enhanced functionality to curate peptide identifications into an MS/MS peptide spectral library for all of our protein database search identification results.

## Introduction

Proteomics is a key method for advancing our understanding of biological processes and systems. Making this technology accessible to the biological community is critically important [1]. The rapid growth of mass spectrometry (MS) data in proteomics research has necessitated the creation of new bioinformatics tools and databases to efficiently pull together diverse sets of analyses. With the growing use of high-throughput proteomics technologies in life science research, there is a call for “democratizing” proteomics data [2], that is, making the source data in scientific publications available to the readers. Although making MS data publicly available has still not been widely mandated by journals as a requirement for publication, a number of public databases have been created for accepting data submissions (post-publication or as part of the publication process) from the proteomics community. As reviewed by Vizcaíno et al. [3], these databases include the Global Proteome Machine (GPM) [4], Proteomics Identifications database (PRIDE) [5], and PeptideAtlas [6]. The 2014 NAR database registry provides a more comprehensive list of public proteomics resources (http://www.oxfordjournals.org/nar/database/cat/10) such as Model Organism Protein Expression Database (MOPED) [7] and Plasma Proteome Database (PPD) [8].

While the importance of sharing proteomics data broadly has been emphasized [9], the kind and format of data and metadata to share (in addition to when to share the data) have been an active topic of discussion in the proteomics community. This has led to a number of proteomics data standard initiatives (http://www.psidev.info) [10]. One of these initiatives is the Minimum Inofrmation about A Proteomics Experiment (MIAPE) [11], whose goal is to specify the information necessary to interpret the results of the proteomics experiment unambiguously and to potentially reproduce the results of the experiment.

As the amount of public proteomics data increases rapidly, concerns have been raised regarding data quality. For example, Schaab et al. [12] have pointed out the issue of data quality existing in public proteomics databases due to heterogeneous sources. This makes data comparison and integration difficult across proteomics experiments conducted independently by different research groups. To address issues such as these, we developed Yale Protein Expression Database (YPED; version 1.0) [13] as a uniform system for collecting proteomic data derived from multiple samples that have been submitted by hundreds of investigators for analysis in the Keck Foundation Biotechnology Resource Laboratory at Yale University. This uniformity of sample entry into YPED ensures that only precise and high quality data, *e.g*., protein identification results filtered with 1% false discovery rate (FDR), are curated for future proteomic experimentation. Subsequently, other laboratories have implemented data filtering models such as MaxQB [12] and Panorama [14] (http://proteome.gs.washington.edu/software/skyline). In addition to discovery proteomics, targeted proteomic assays have become more common [15]. Therefore, there is a growing need for proteome data to be well curated into MS/MS spectral libraries and for more integrative multiple reaction monitoring (MRM)/selective reaction monitoring (SRM) tools to be developed [15]. Several public libraries already exist, such as PeptideAtlas [6], SRMAtlas [16], National Institute of Standards and Technology (NIST) Libraries of Peptide Tandem Mass Spectra (http://peptide.nist.gov/), GPMDB [4], and the PeptideAtlas SRM Experiment Library (PASSEL) [17]. However, these libraries often require expert user intervention to generate MRM/SRM transition lists.

**Figure 1:**
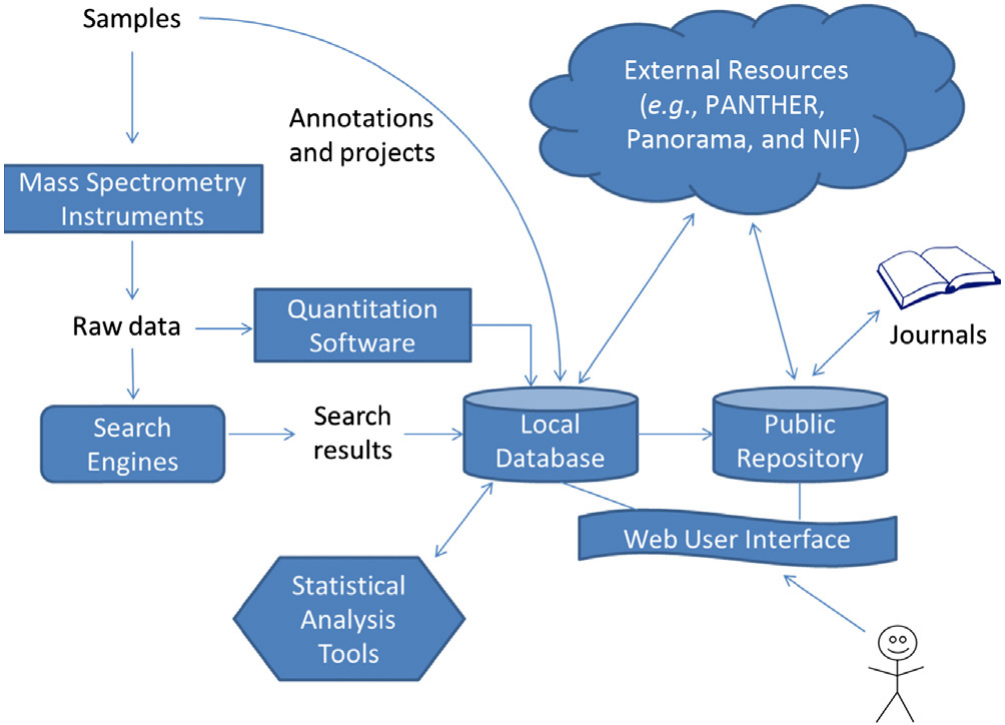
Workflow diagram summarizing YPED system components and their relationships

In light of these challenges, we present here a significantly-enhanced version of YPED, an open-source proteomics suite and database [13]. Figure 1 displays the main components of the YPED system. In contrast to laboratory-specific and community-based proteomics databases, YPED is unique in providing a comprehensive workflow that extends from sample submission through a web user interface, which provides immediate access to newly-acquired data, to an integrated suite of biostatistical and bioinformatics tools for analyzing the resulting mass spectrometric proteomics data. On the other hand, YPED consists of both a local database and a public repository that provides access to published and anonymous results. The wide range of data access privileges of YPED enables it to meet the needs of individual, multiple collaborative, and core laboratories. It supports multiple MS instruments and search engines. It also supports quantitation of labeled and label-free proteomics data. Sample/project annotations and search results stored in the database can be queried and viewed via a web user interface. We have also developed and integrated a suite of statistical analysis tools to enhance the quality and visualization of data. In addition, the YPED system is interoperable with a number of external resources to leverage proteomics databases and tools created by other groups. The source code of the YPED system can be downloaded from http://yped.med.yale.edu/yped_dist/. A demo account with Username as yped_demo and Password as yped_demo contains representative data results.

YPED’s increasingly important role in biomedical research is highlighted by its usage statistics. As of January 12, 2015, YPED contained 18,985 datasets from 1654 users in the laboratories of 702 principal investigators at more than 300 institutions around the world. These datasets contained liquid chromatography (LC)-MS/MS analyses from 3,997,386 distinct peptides derived from 929,665 distinct proteins. YPED’s spectral library contains spectra from 340,449 distinct human peptides, which are more than the 293,000 non-redundant spectra used by Kim et al. [18] to map the human proteome. YPED’s spectral library contains ⩾2 distinct peptides from 19,327 human, 16,154 mouse, 7661 rat, 6007 yeast, and 4080 *Escherichia coli* proteins, respectively.

## Methods

### User statistics and summary

YPED is a web-accessible, password-protected database for managing high-throughput proteomic analyses. For a comprehensive, current usage statistics report for YPED that is updated daily please visit: https://yped.med.yale.edu:8443/yp_results/QDSTATS_report.do. We have extended YPED’s functionality to keep in step with rapidly-evolving MS and proteomic technologies. The initial report (YPED version 1.0) [13] described analysis requisition, result reporting and sample comparison for multi-dimensional protein identification technology (MudPIT) [19], difference gel electrophoresis (DIGE) [20], and isotope-coded affinity tag (ICAT) labeled [21] samples. In addition, YPED now includes modules for LC–MS peptide and protein identifications (LC–MS/MS), multiplexed isobaric tagging technology (iTRAQ [22] and tandem mass tag (TMT) [23]), stable isotope labeling by amino acids in cell culture (SILAC) [24], LC–MS/MS label-free quantitation [25] (Skyline and Progenesis), and scoring for phosphopeptide localization (Mascot Delta Score (MD-score) [26] and PhosphoRS [27]). Using the discovery proteomic results, we have built a MRM/SRM targeted proteomics pipeline that includes an MS/MS spectral library. The peptide sequences in the spectral library have been compared via protein BLAST [28] against Swiss-Prot and TrEMBL databases [29] to determine if these sequences are unique to a specific protein and organism.

**Figure 2:**
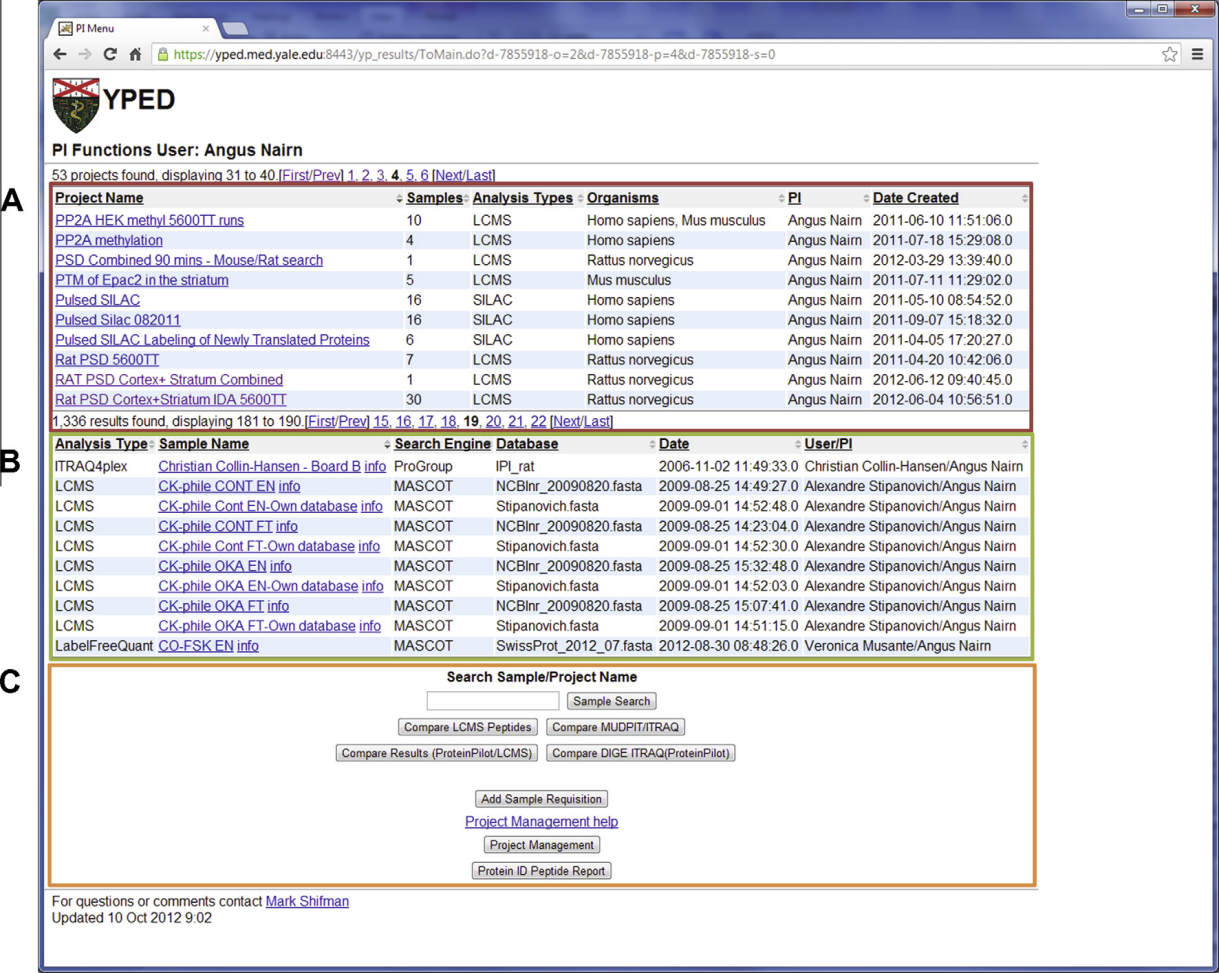
**YPED PI/User main menu** The main menu is broken down into three sections which are outlined in red (**A**), green (**B**), and orange (**C**) boxes, respectively. The red section (A) contains the project listing that is made up of collections of individual sample results. The green section (B) contains a list of all individual sample results. The orange section (C) highlights all the user options. Users can search for sample, perform peptide/protein sample comparative analysis, initiate new sample requisitions, perform project management, and search the protein/peptide spectral library.

Individual researchers can access their data through a simple user interface (Figure 2). Principal investigators (PIs) can also access all datasets generated by staff from within their laboratories. Individual experimental results are listed as samples, which can then be grouped into projects to help researchers keep track of different stages of their project. Each sample contains the experimental fields necessary to meet the MIAPE sample guidelines, including information such as sample preparation protocols, proteomics instrumentation and methodology, so results can be reproduced and compared. Not only does this data organization/annotation enhance data sharing, but it also facilitates the publication process. A publication can be associated with one or multiple samples and/or projects. Researchers can view, subset and download their data through the secure web interface. There are also proteomics core “superuser” accounts (Figure S1) that allow multiple staff in one or more proteomics cores to upload MS data. In addition, YPED also features modules for sample submission, tracking, and billing. The “regular” user interface (Figure 2) contains three sections: the project listings, sample listings, and user functions such as search, sample requisition, and project management. The “superuser” interface (Figure S1) provides the ability to carry out many additional options such as sample submission, project management, sample tracking, data import, sample administration, and user billing. Additionally, within projects, superusers or users can organize and provide additional documentation to their datasets by linking raw data and/or associated documents (*e.g.*, PDF and PowerPoint files).

### System implementation

YPED is available as an open-source package. The web application is written in Java using Struts (version 1.3.10). The web server is configured using Tomcat 7.0.20 and connects to an Oracle database (version 11g). It also connects to a Windows-based file server through file transfer protocol (FTP). The source code, javadoc and oracle schema can all be downloaded from the web page (http://yped.med.yale.edu/yped_dist/).

## Results

### LC–MS protein identification

**Figure 3:**
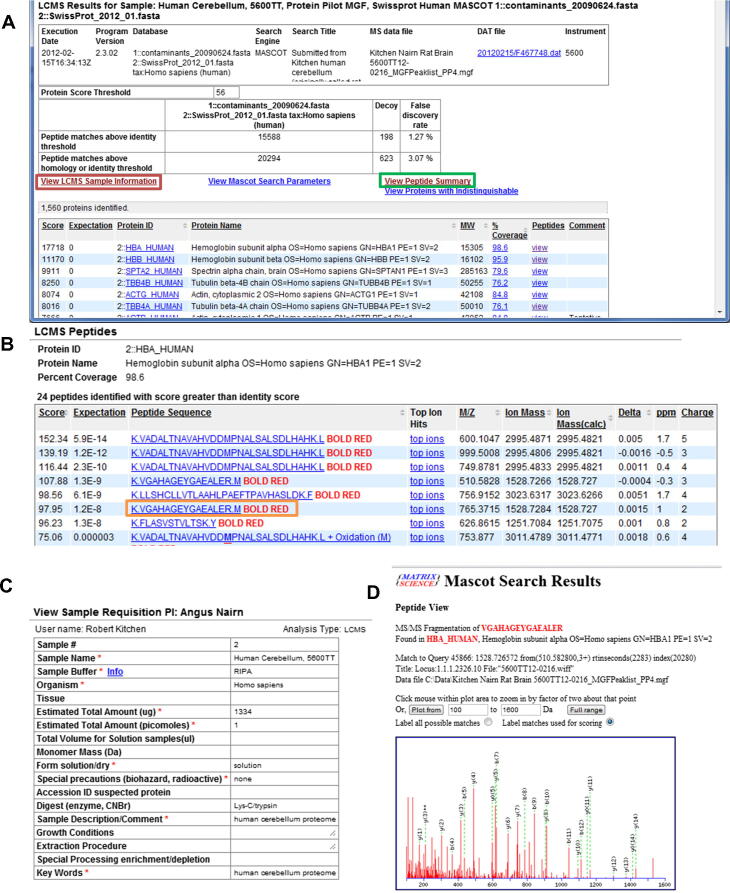
**YPED LC–MS result page** **A**. Main LC–MS result page. The header contains summary information such as sample name, date, Mascot version, sequence database, and mass spectrometer used for analysis. It also displays the Mascot protein ID threshold and FDR statistics. Below the header information are four hyperlinks that navigate the user to ancillary information. The first hyperlink outlined in the green box goes to the peptide summary page (**B**). The second hyperlink outlined in the red box provides a sample description and information page (**C**). The other two hyperlinks (navigation results not shown) provide details on the Mascot search parameters used for database searching and a summary for indistinguishable proteins, respectively. The peptide summary page (B) displays information on all the protein identifications and also contains additional hyperlinks for viewing each individual MS/MS spectra. Navigating through the orange button highlighted above, users are directed to a Mascot peptide view page (**D**).

Version 1.0 of YPED supported ProteinProphet (protXML) and PeptideProphet (pepXML). In the extended version we added an LC–MS module to include results from Mascot (Matrix Science Inc.) search (current version 2.4.0) and ProteinPilot. Mascot results are imported after transformation into an XML file employing the Mascot script, export_dat_2.pl. YPED also supports ProteinPilot (Paragon)∗.group result files that have been converted to an XML document. We then developed an XML schema definition (ProteinPilot4.xsd) that enables either of the resulting XML files to be parsed and loaded into YPED using JAXB (http://jaxb.java.net/) and Java StAX API (http://stax.codehaus.org/). These results can be viewed via the web and include FDRs, the proteins identified with scores and coverage maps, and peptides identified for each protein with attendant peptide scores (Figure 3). Data are presented via a browser in tables where summary facts can be conveniently browsed using hyperlinks, enabling users to drill all the way down to the MS/MS data. Users have the option of additionally processing their protein identification data through ProteinProphet (protXML) and PeptideProphet (pepXML) and displaying the combined results (Figure S2). YPED also contains additional protein identification information such as the exponentially-modified protein abundance index (emPAI) [30], which enables estimation of absolute protein amount within a complex proteome sample. Although the emPAI results are not displayed on the main LC result page, they are contained in the exported Excel spreadsheet (Figure S3).

### Label-based quantitative analysis

iTRAQ [22] and TMT reagents [31] allow multiplexing of protein samples and produce identical MS spectra but label specific reporter fragment ions for the multiple versions of the labeled peptide. YPED currently supports mass spectrometric data processing with either ProteinPilot [32] (AB Sciex Inc.) or Mascot software. Both packages perform protein identification and peptide reporter ion quantitation. Protein and peptide data results from ProteinPilot are exported as comma-delimited text files (.csv format) and imported into YPED. For Mascot iTRAQ/TMT quantitation results, both the protein identifications and peptide reporter ions are imported as described in the above LC–MS protein identification section (Figure S4).

SILAC [24] studies can be processed by initial database searching with Mascot and then using the quantitation toolbox in Mascot Distiller (Matrix Science Inc.). The resulting Mascot distiller XML output is then processed with JAXB and the Java StAX API before insertion into YPED. The web results page displays the LC–MS results along with the heavy/light ratios and SILAC peptides.

### Label-free quantitative analysis

**Figure 4:**
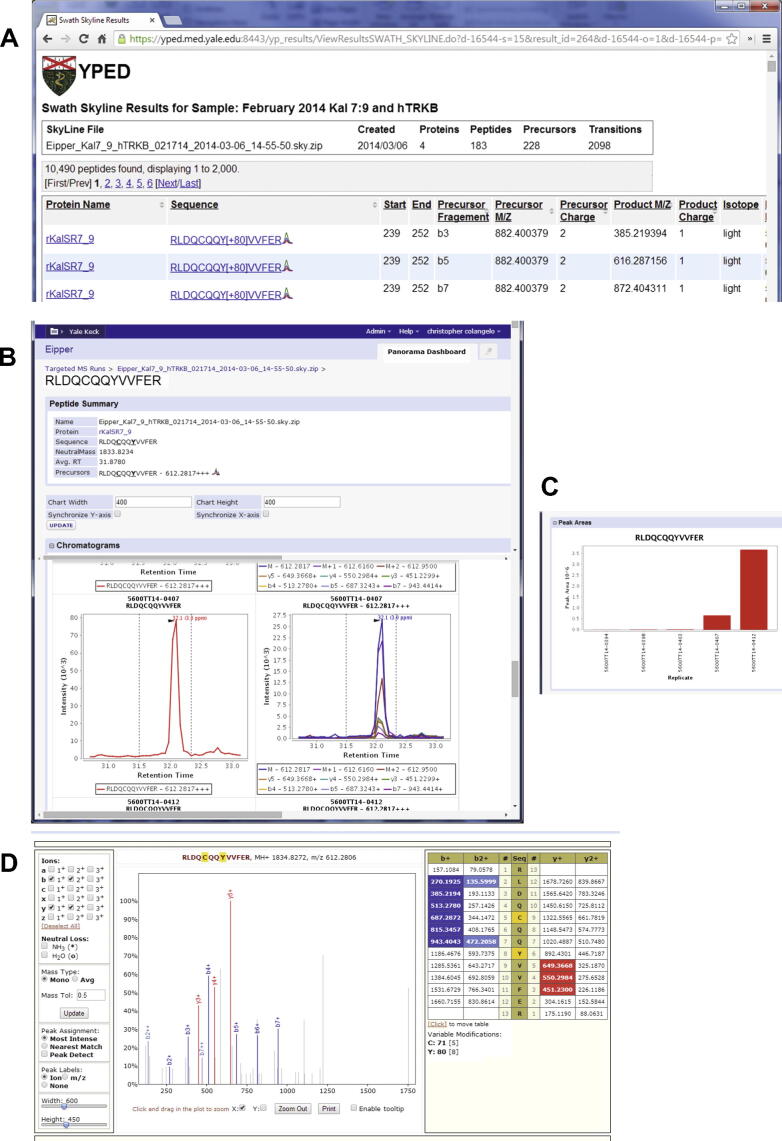
**Skyline Label-free SWATH results in YPED** **A**. Clicking on the Sequence hyperlinks brings the user to the Panorama data repository. **B**. Panorama web interface shows one of the peptide sequences for the associated Skyline document. The web interface provides a more detailed view for the peptide that includes chromatograms for the precursors in all the replicates. Graphs show the peak areas (**C**) for the peptide measured in individual replicates and the associated MS/MS spectra from the corresponding spectral library (**D**). The source document can be downloaded via a DOWNLOAD link for viewing in Skyline.

**Figure 5:**
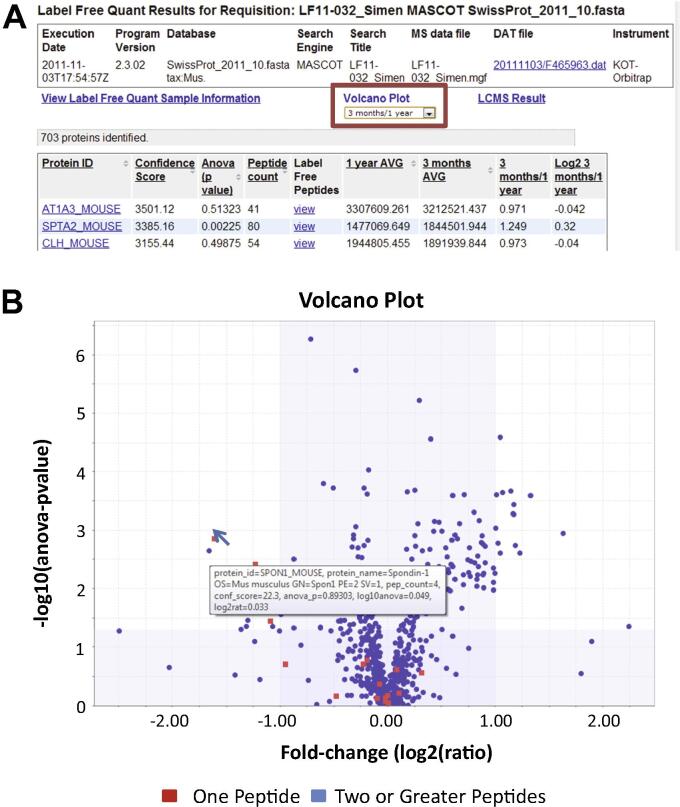
**Screenshot of the Label-free quantitation data results** YPED features data from LC–MS based label-free quantitative proteomics with integrated data uploaded from Progenesis LC–MS software (Nonlinear Dynamics Inc.). The user can visualize quantitation at the peptide and protein level. **A**. Clicking on the hyperlinked “Volcano Plot” option in the red box brings up the protein level, annotated Volcano plot shown in (**B**). Navigating the mouse over the Volcano plot (B) provides a pop-up box containing a detailed description of protein fold change and *P* values for each of the 703 proteins depicted in the Volcano plot with red (one peptide) or blue (two or more peptides) dots. (For interpretation of the references to colour in this figure legend, the reader is referred to the web version of this article.)

LC–MS/MS label-free quantitation data can be processed with either Skyline or Progenesis LC–MS software (Nonlinear Dynamics, LLC), with Skyline also enabling analysis of LC–SWATH datasets. For Skyline, the peak integration results are uploaded to Panorama and also exported to a comma delimited text (∗.csv) file. The text file is then uploaded to YPED, where these results are merged to generate a report table as shown for SWATH data in Figure 4. This report contains protein ID, peptide sequence, isotope dot product, and quantitation values. In addition, YPED contains links to the stored chromatograms on Panorama, where users can visualize their Skyline peak integration results (Figure 4). Label-free Progenesis LC–MS results are exported to Excel, parsed with the POI Java library (http://poi.apache.org/), and inserted into YPED. The Mascot search results are imported as described above. YPED merges both these results to generate a web report table (Figure 5) that contains protein ID, confidence scores, quantitation values, ratios and ANOVA *P* values with options for generating a Volcano plot of the results. In addition, individual peptide identifications can be conveniently browsed using hyperlinks, enabling users to drill all the way down to the MS/MS data.

### Phosphoprotein analysis

**Figure 6:**
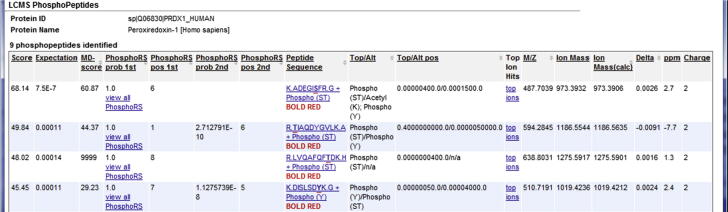
**Screenshot of phosphopeptide localization results** Information on a subset of the identified phosphopeptides for PRDX1_human is shown, which includes peptide sequence, Mascot score, MD-score, and phosphoRS score for each site identified. These results enable researchers to confidently assign a phosphorylation site to any MS/MS spectra. Thus, identified phosphopeptides from any YPED experiment can then be further queried to view the probability that a specific phosphorylation site is actually phosphorylated using either MD-score [26] and/or phosphoRS [27] scoring algorithms and thereby have high confidence that the site localization is correct.

To leverage newly-developed tools that help to identify sites of peptide phosphorylation, YPED was upgraded to include both phosphoprotein filters and phosphopeptide scoring algorithms to aid in site localization analysis. These upgrades enable researchers to automate phosphopeptide site localization on large LC–MS datasets and have high confidence that the site assignments are correct. To access the phosphoprotein filter from the LC–MS, SILAC, or label-free quantitation results, users simply click the hyperlink, “View PhosphoProteins”, which then brings up a web page that displays a listing of the phosphoproteins identified and the number of phosphopeptide matches for each protein. Further navigation can be done by clicking the “view” hyperlink under the phosphopeptide column in the table, after which YPED will then generate a table containing rows of identified phosphopeptides with each phosphorylated amino acid underlined and with columns containing the associated MD-score [26], PhosphoRS [27] probability score, *m/z*, ion mass, mass accuracy (ppm), and peptide charge (Figure 6).

### Comparative analysis

Tools have been added to facilitate downstream sample comparison and to assess the distribution of biological functions (through a remote query to PANTHER [33]) among the identified proteins in a sample. For downstream analysis, researchers can compare samples based on peptide or protein content, or cross-compare the proteins from various analyses such as comparing a MudPIT to an iTRAQ analysis. A pairwise analysis on each sample is performed and the results are listed in a table format with distinct peptides/proteins in each sample and the peptides that overlap between all samples (Figure S5).

### Targeted proteomics

An entire targeted proteomics pipeline has been integrated into YPED, which enables utilization of our custom peptide spectral library database (see below) to facilitate peptide and MRM/SRM transition selection for global targeted proteomic analysis, tools for method export, and an interface for collation of quantitation data results and review. Specifically, transitions and retention times can be rapidly retrieved from database search results to guide the validation of complex large-scale discovery studies by MRM-based targeted proteomics. To generate a targeted proteomics experiment, users first query the entire YPED spectral library using the “Protein ID Peptide Report” search tool, which has filters for protein accession numbers, protein names, peptide sequences, and gene symbols. YPED then displays the search results in a browser, where users select peptides to add to a targeted proteomics experiment list. When the list is finalized, YPED automatically filters proteins/peptides on the server without the need for expert user intervention, thereby maximizing productivity. YPED uses the following criteria for filtering. First, peptide scores have to be greater than or equal to the identity score. Second, proteins must have three or more peptides. Third, peptides that match 1 protein in the given species specific BLASTP [28] search are kept. Fourth, peptides containing methionine residues are excluded. Finally, the remaining peptides are sorted based on their number of occurrences in YPED with the top peptides being chosen for downstream MRM/SRM analysis. After peptide selection, the highest ion intensities are selected as transitions for downstream MRM/SRM analysis. These MRM/SRM transitions along with their retention times are exported as a tab-delimited file (tsv) and then used to populate a targeted mass spectrometer method file.

### Spectral library for downstream MRM/SRM assay development

The spectral library is generated by first taking each Mascot search result and filtering it at 1% FDR. Then all the unique LC–MS peptide identifications with Mascot peptide scores greater than homology and 5–30 amino acids in length are compared to the Swiss-Prot database using a protein BLAST search [28]. Table 1 shows a summary of the BLASTP results for five model organisms commonly used in proteomic analyses. The BLASTP results are stored in YPED as a table which includes the number of observations per peptide and each individual observation. After BLAST analysis, we filtered the number of proteins to 19,327 for human, 16,154 for mouse, and 7661 for rat with two or more distinct peptides per protein. These results are then used to verify that a given set of candidate peptides are unique to a protein when determining targeted (SRM/MRM) candidates for future assays. We also have implemented the ability to export either individual samples or a project (series of samples) from Mascot search files to BiblioSpec format [34] utilizing Blibbuild. The resulting spectral libraries can be utilized in searching MS/MS spectra [35] or for Skyline.

**Table 1:** YPED spectral library BLAST results (UniProtKB/SwissProt Database)

**Species**	**Blast protein ount**	**Blast peptide ount**
*E. coli*	4080	48,003
Yeast	6007	75,253
Rat	7661	154,580
Mouse	16,154	287,242
Human	19,327	340,449

*Note:* Proteins and peptides are filtered prior to being added to our spectral library. Protein filtering criteria were as follows; for a protein to be identified, it must contain multiple matches to more than one peptide from the same protein and their peptides must have a Mascot score greater than or equal to the homology score.

### Public repository

**Figure 7:**
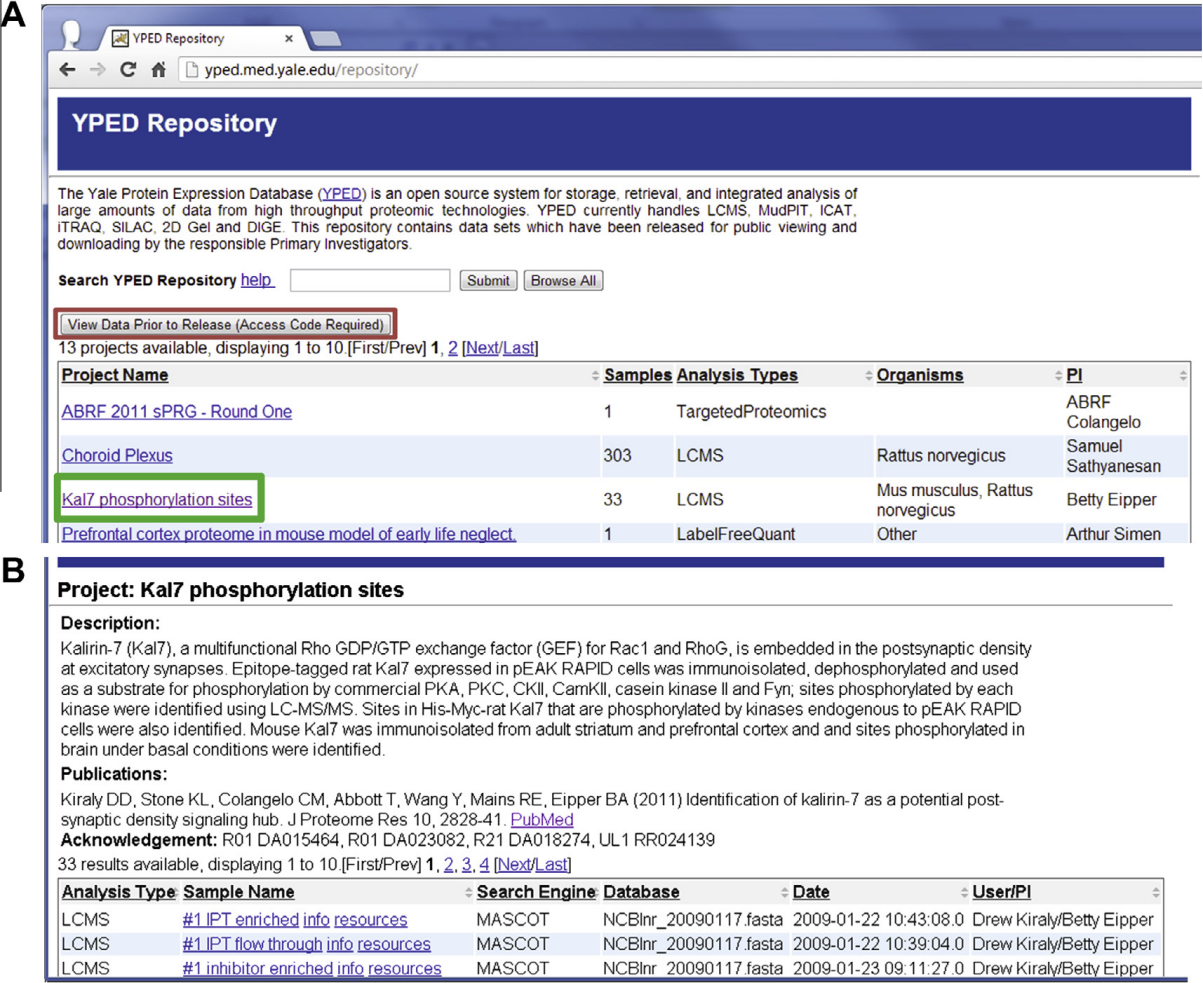
**YPED Repository** Data associated with a published paper can be released to a publicly-accessible repository called the YPED Repository (**A**). Private (anonymous) access by reviewers to data associated with manuscripts under review can be given using an access code and data can be accessed by navigating the red hyperlink in the YPED repository page (A). Hyperlinking through the green outlined box navigates to an individual project summary page (**B**), which contains a project description, citation, acknowledgements, and a table with individual sample results. In the sample results table, users can further navigate using the “info” hyperlink to view sample preparation information or the “resources” hyperlink to download zipped data files (*e.g.*, Mascot mgf files, Mascot dat files, and mzML files).

We have developed a publicly-accessible YPED repository to further increase accessibility to YPED’s proteomics data (http://yped.med.yale.edu/repository) (Figure 7). It contains the results of projects that have been released for public viewing by the principal investigators along with raw data from the samples. To broaden the visibility and interoperability, we have also released the project results to the Neuroscience Information Framework (NIF) federated data repository (https://www.neuinfo.org/mynif/databaseList.php). This allows YPED to be integrated with a wide variety of neuroscience databases to enhance its support of neuroproteomics research. The YPED repository also has an access code provision for viewing results prior to public release. This feature is useful for making the results available to reviewers and collaborators who do not have YPED access.

The repository provides a query interface to search anonymous results based on protein IDs/names, peptide sequence and gene symbols. Figure S6 shows a portion of the search results for a protein whose ID is KCC2G_HUMAN. The search returns 51 distinct peptides above the peptide score threshold.

## Discussion

To tackle the huge data challenges posed by high-throughput LC/MS/MS proteomics datasets, we have assembled a team from a broad range of disciplines including bench scientists, clinicians, computer scientists (with database and high-performance computing expertise), bioinformaticians, biostatisticians, and proteomics technologists. Such a multidisciplinary approach was a key to developing YPED into a user-friendly, scalable, evolvable and sustainable resource. The resulting YPED is an integrated suite of tools designed to cover a broad spectrum of techniques for quantitative proteomics (discovery and targeted proteomics; and labeled and label-free quantitation). It captures data produced by a wide range of MS instruments and technologies, and presents them via the web as a set of relevant results that are understandable for non-specialists.

YPED implements a wide range of data access privileges associated with different user types including core laboratory users, researchers (PIs and their laboratory members), and public users. One advantage of this approach is that it allows data sharing at different levels. For example, researchers can share their data within a specific laboratory and/or between laboratories (possibly located at different institutions). Core facility users can help individual laboratories to populate data in YPED as they have read/write access to the laboratories they work with. YPED was started with one core facility (Keck Foundation Biotechnology Resource Laboratory at Yale). Recently, we have added another core proteomics facility, West Campus Analytical Chemistry Core that is part of the West Campus expansion at Yale. In the future, we may be able to add core facilities beyond Yale who are willing to adhere to the same high standards of data quality (*e.g.*, 1% FDR filtered protein identification results). In addition to security, the different user roles facilitate collaboration in a trusted environment.

The first version of YPED [13] only supported a few technologies, but as mass spectrometric methods have evolved we extended YPED (version 2.0) to handle these new data types. Ongoing work includes integrating YPED to handle additional quantitative techniques and programs (*e.g.*, Maxquant and data-independent analysis such as SWATH [36]) and to update as new instruments are obtained. We also would welcome the opportunity to expand YPED’s linkage to external databases/knowledge bases such as PRIDE or PeptideAtlas. In addition to PANTHER, we will enable YPED to incorporate information from pathway and protein network resources such as KEGG [37], Reactome [38], and STRING [39].

While we will continue to address the needs of individual laboratories, we also will increase our interaction with the proteomics community, such as the Association of Biomolecular Resource Facilities (ABRF; http://www.abrf.org/) and Human Proteome Organization (HUPO; http://www.hupo.org/), to help promote the use and development of standards (*e.g.*, HUPO-PSI [40]) for exchanging data with other major proteomics databases (*e.g.*, PRIDE, GPM, PASSEL and PeptideAtlas). For example, in addition to producing our spectral libraries in BiblioSpec format, we are working to support mzIdentML (http://www.psidev.info/mzidentml), since a growing number of tools support these standardized formats. As biomedical ontologies have increasingly been applied to proteomics databases such as PRIDE, we will also explore the use of ontologies to standardize proteomic data annotation and enable ontologically-based data integration. Finally, we have created a virtual machine for YPED that greatly increases the flexibility and ease of future deployment of YPED to other institutions or into a shared infrastructure (*e.g.*, in the cloud) accessed by multiple institutions.

## Author’s contributions

KHC, PLM and KRW supervised the study. CMC oversaw the design of YPED and MS wrote the source code for YPED. CMC wrote the manuscript and ACN edited the manuscript. KLS, NJC, EEC, TTL, TW, RDB, CB, and JR all contributed ideas for improving YPED. All authors read and approved the final manuscript.

## Competing interests

The authors declared that there are no competing interests.

## Supplementary material

Figure S1 **Screenshot of the SuperUser interface** The “superuser” can perform sample submission, project management, sample tracking, data import, sample administration, and user billing. The superuser can also query both the spectral library and synthetic peptide library as well as generate MRM-based assays using YPED’s targeted proteomics/small molecule quantitation workflow. The superusers can also access the administrative functions page, which enables them to add/edit users, verify users, and generate access codes for pre-release data to the YPED repository. In addition, the administrative page also provides database metric and usage statistics, such as the number of samples run and total number of proteins identified.

Figure S2 **Screenshot of the LC-MS results page combining Mascot and ProteinProphet results A.** Main LC-MS result page. Header contains summary information such as sample name, date, Mascot version, sequence database, and mass spectrometer used for analysis. It also displays the Mascot protein ID threshold and FDR statistics. Below the header information are four hyperlinks that navigate to ancillary information. The hyperlink outlined in the red box takes the user to the ProteinProphetProbability Cutoff *vs.* False Positive Error Rate Table shown in (**B**). The hyperlink in the green box from the Main LC-MS result page (A) displays the peptide summary page (**C**), which contains the individual peptide Mascot score, Peptide sequence, *m/z*, ppm error, parent ion charge and PeptideProphet probability value

Figure S3 **LC-MS protein identification export table from YPED** The protein export table contains additional information such as emPAI that is not shown in the main YPED table.

Figure S4 **Screenshot of the Mascot TMT quantitation results A.** Header contains summary information such as sample name, date, Mascot version, sequence database, mass spectrometer used for analysis, as well as the Mascot protein ID threshold and FDR statistics. **B.** Below the header information are five hyperlinks that navigate to ancillary information. The first hyperlink entitled “View TMTsixplex Sample Information” displays the sample and TMT tagging information. The second and third hyperlinks entitled “View Mascot Search Parameters” and “View Mascot Quantitation Parameters” display the search and integration parameters used for the analysis, respectively

Figure S5 **Results of using comparison tool in YPED A.** The first panel in the upper left hand corner shows the results of a pairwise analysis of three iTRAQ samples in table format. **B.** Clicking on the hyperlink “RESULT_1 distinct” (boxed in red) shows the proteins and corresponding iTRAQ ratios that were uniquely identified in Sample 1. **C.** Clicking on the link “RESULT_1 x RESULT_2 pairwise intersection” (boxed in green) provides the proteins shared between Sample 1 and Sample 2. **D.** Clicking on the link “Proteins common to all samples” (box in orange) provides the proteins shared between all replicates.

Figure S6 **YPED repository A**. Search interface. **B.** Search results for a protein with the ID KCC2G_HUMAN.
