# Supplementary figures and images for: YPED: An Integrated Bioinformatics Suite and Database for Mass Spectrometry-based Proteomics Research
Source: Genomics Proteomics Bioinformatics. 2015 Feb 21;13(1):25–35. doi: 10.1016/j.gpb.2014.11.002 (PMC4411476; doi:10.1016/j.gpb.2014.11.002)

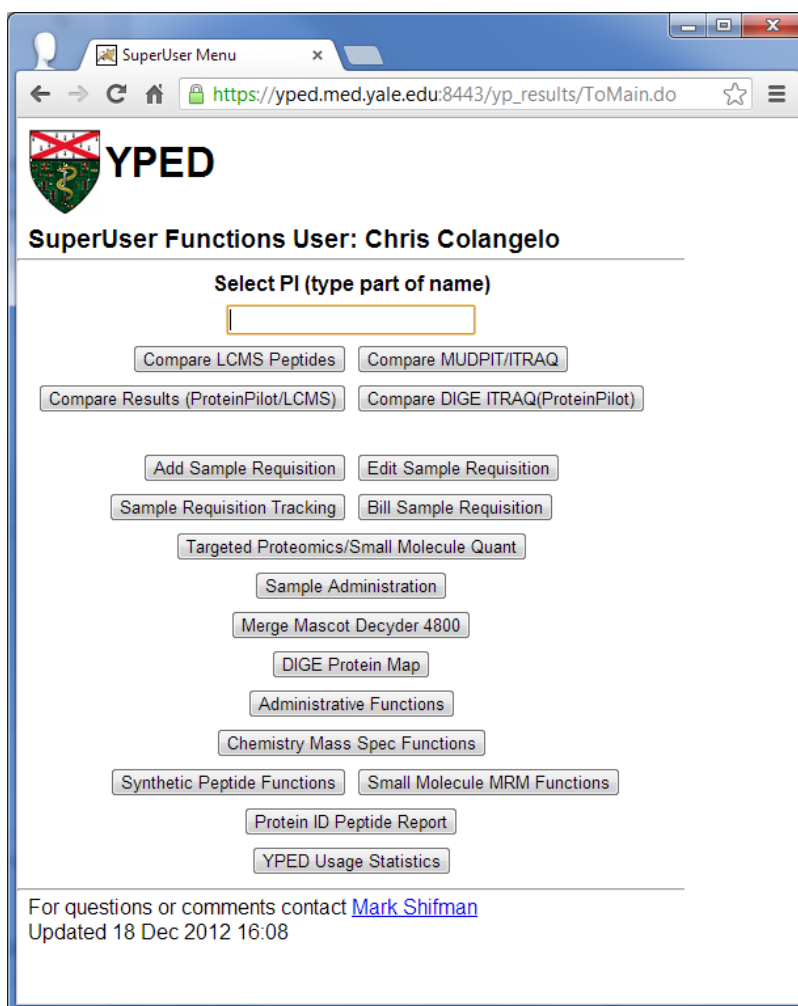

**Supplemental Figure 1.**

Supplement: Figure S1 — Screenshot of the SuperUser interface The “superuser” can perform sample submission, project management, sample tracking, data import, sample administration, and user billing. The superuser can also query both the spectral library and synthetic peptide library as well as generate MRM-based assays using YPED’s targeted proteomics/small molecule quantitation workflow. The superusers can also access the administrative functions page, which enables them to add/edit users, verify users, and generate access codes for pre-release data to the YPED repository. In addition, the administrative page also provides database metric and usage statistics, such as the number of samples run and total number of proteins identified. [file mmc1.pdf]

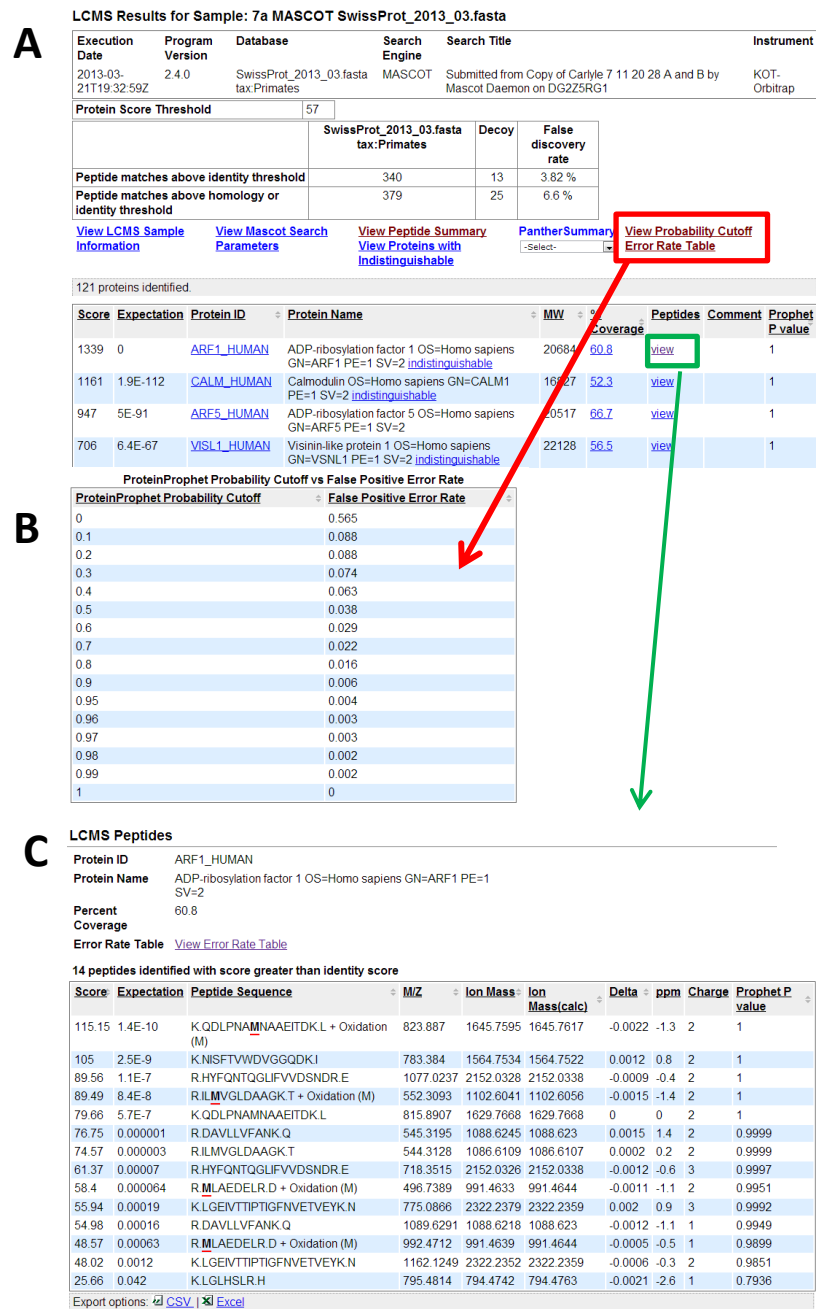

Supplemental Figure 2.

Supplement: Figure S2 — Screenshot of the LC-MS results page combining Mascot and ProteinProphet results A. Main LC-MS result page. Header contains summary information such as sample name, date, Mascot version, sequence database, and mass spectrometer used for analysis. It also displays the Mascot protein ID threshold and FDR statistics. Below the header information are four hyperlinks that navigate to ancillary information. The hyperlink outlined in the red box takes the user to the ProteinProphetProbability Cutoff vs. False Positive Error Rate Table shown in (B). The hyperlink in the green box from the Main LC-MS result page (A) displays the peptide summary page (C), which contains the individual peptide Mascot score, Peptide sequence, m/z, ppm error, parent ion charge and PeptideProphet probability value [file mmc2.pdf]
