# Supplementary material for: YPED: An Integrated Bioinformatics Suite and Database for Mass Spectrometry-based Proteomics Research
Source: Genomics Proteomics Bioinformatics. 2015 Feb 21;13(1):25–35. doi: 10.1016/j.gpb.2014.11.002 (PMC4411476; doi:10.1016/j.gpb.2014.11.002)
Supplement: Figure S3 — LC-MS protein identification export table from YPED The protein export table contains additional information such as emPAI that is not shown in the main YPED table. [file mmc3.pdf]

Score	Expectation	Protein ID	Protein Name	MW	% Coverage	Comment	empai	Prophet P value
1339	0	ARF1_HUMAN	ADP-ribosylation	20684	60.8		20.36	1
1161	1.90E-112	CALM_HUMAN	Calmodulin OS=H	16827	52.3		202.74	1
947	5.00E-91	ARF5_HUMAN	ADP-ribosylation	20517	66.7		16.42	1
706	6.40E-67	VISL1_HUMAN	Visinin-like prote	22128	56.5		13.33	1
690	2.90E-65	COF1_HUMAN	Cofilin-1 OS=Hom	18491	58.4		28.92	1
643	1.40E-60	ARF4_HUMAN	ADP-ribosylation	20498	46.7		4.8	1
547	5.30E-51	MBP_HUMAN	Myelin basic prot	33097	21.7		2.46	1
518	4.40E-48	HPCA_HUMAN	Neuron-specific c	22413	44.6		10.27	1
486	6.70E-45	HPCL1_HUMAN	Hippocalcin-like p	22299	49.7		10.4	0.9842
480	3.00E-44	NCALD_HUMAN	Neurocalcin-delta	22231	43		8.41	1
474	1.00E-43	HPCL1_PONAB	Hippocalcin-like p	22211	42	Tentative 1 s	8.41	0.9946
393	1.20E-35	COX2_MACMU	Cytochrome c oxi	25474	22		3.17	1
295	8.20E-26	ARPC3_HUMAN	Actin-related pro	20533	23.6		2.74	1
283	1.30E-24	PEBP1_HUMAN	Phosphatidyletha	21044	35.3		1.92	1
279	3.20E-24	DUS3_HUMAN	Dual specificity p	20465	40.5		2.02	1
244	1.00E-20	ML12A_HUMAN	Myosin regulatory	19781	34.5		2.93	1
219	3.20E-18	NDUS7_GORGO	NADH dehydroge	23471	26.3		1.17	1
205	8.70E-17	SKP1_HUMAN	S-phase kinase-as	18646	24.5		1.63	1
202	1.50E-16	ARPC4_HUMAN	Actin-related pro	19654	28		2.15	1
199	3.10E-16	PPIB_HUMAN	Peptidyl-prolyl ci	23728	26.4		2.14	1
195	8.60E-16	TBB3_HUMAN	Tubulin beta-3 ch	50400	6.9		0.31	0.9996

Supplemental Figure 3.
