# Supplementary material for: YPED: An Integrated Bioinformatics Suite and Database for Mass Spectrometry-based Proteomics Research
Source: Genomics Proteomics Bioinformatics. 2015 Feb 21;13(1):25–35. doi: 10.1016/j.gpb.2014.11.002 (PMC4411476; doi:10.1016/j.gpb.2014.11.002)
Supplement: Figure S4 — Screenshot of the Mascot TMT quantitation results A. Header contains summary information such as sample name, date, Mascot version, sequence database, mass spectrometer used for analysis, as well as the Mascot protein ID threshold and FDR statistics. B. Below the header information are five hyperlinks that navigate to ancillary information. The first hyperlink entitled “View TMTsixplex Sample Information” displays the sample and TMT tagging information. The second and third hyperlinks entitled “View Mascot Search Parameters” and “View Mascot Quantitation Parameters” display the search and integration parameters used for the analysis, respectively [file mmc4.pdf]

A

YPED

TMTsixplex Results for Sample: TMTsixplex demo MASCOT SwissProt_2013_02.fasta

Execution Date	Program Version	Database	Search Engine	Search Title	Instrument
2013-02-15T18:32:38Z	2.4.0	SwissProt_2013_02.fasta tax:All entries	MASCOT	HCD_Colangelo_TMT	Orbi

Protein Score Threshold	70

	SwissProt_2013_02.fasta tax:All entries	Decoy	False discovery rate
Peptide matches above identity threshold	334	6	1.8 %
Peptide matches above homology or identity threshold	607	15	2.47 %

B

View TMTsixplex Sample Information

View Mascot Search Parameters

View Mascot Quantitation Parameters

View Peptide Summary

View Proteins with Indistinguishable

PantherSummary

-Select-

31 proteins identified.

Score	Expectation	Protein ID	Protein Name	MW	% Coverage	Peptides	Comment	127/126 Ratio	127/126 N	127/126 SD	127/126 Significance	128/12 Ratio
2060	0	OVAL_CHICK	Ovalbumin OS=Gallus gallus GN=SERPINB14 PE=1 SV=2	48008	58.3	view		0.912	81	[1.156]	---	0.866
1763	0	ALBU_BOVIN	Serum albumin OS=Bos taurus GN=ALB PE=1 SV=4	85223	50.9	view		0.148	99	1.427	*	0.862
1439	0	ADH1_YEAST	Alcohol dehydrogenase 1 OS=Saccharomyces cerevisiae (strain ATCC 204508 / S288c) GN=ADH1 PE=1 SV=5	43011	67	view		2.054	63	[1.116]	---	0.795
1302	3.4E-125	CAH2_BOVIN	Carbonic anhydrase 2 OS=Bos taurus GN=CA2 PE=1 SV=3	33450	66.5	view		5.773	71	1.226	*	0.760

Supplemental Figure 4.
