# Supplementary material for: YPED: An Integrated Bioinformatics Suite and Database for Mass Spectrometry-based Proteomics Research
Source: Genomics Proteomics Bioinformatics. 2015 Feb 21;13(1):25–35. doi: 10.1016/j.gpb.2014.11.002 (PMC4411476; doi:10.1016/j.gpb.2014.11.002)
Supplement: Figure S5 — Results of using comparison tool in YPED A. The first panel in the upper left hand corner shows the results of a pairwise analysis of three iTRAQ samples in table format. B. Clicking on the hyperlink “RESULT_1 distinct” (boxed in red) shows the proteins and corresponding iTRAQ ratios that were uniquely identified in Sample 1. C. Clicking on the link “RESULT_1 x RESULT_2 pairwise intersection” (boxed in green) provides the proteins shared between Sample 1 and Sample 2. D. Clicking on the link “Proteins common to all samples” (box in orange) provides the proteins shared between all replicates. [file mmc5.pdf]

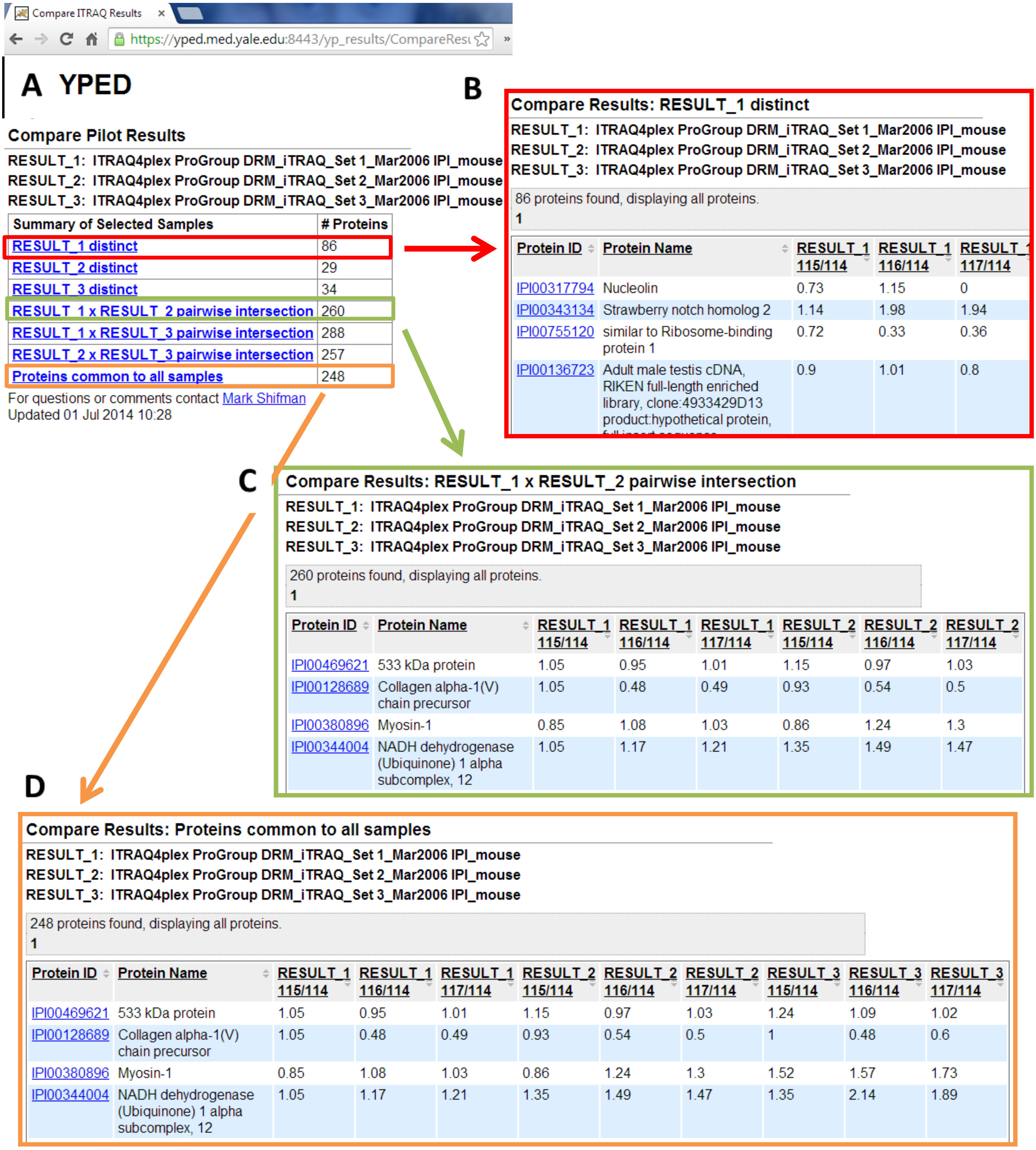

Compare ITRAQ Results

https://yped.med.yale.edu:8443/yp_results/CompareResu

A YPED

Compare Pilot Results

RESULT_1: ITRAQ4plex ProGroup DRM_iTRAQ_Set 1_Mar2006 IPI_mouse
RESULT_2: ITRAQ4plex ProGroup DRM_iTRAQ_Set 2_Mar2006 IPI_mouse
RESULT_3: ITRAQ4plex ProGroup DRM_iTRAQ_Set 3_Mar2006 IPI_mouse

Summary of Selected Samples	# Proteins
RESULT_1 distinct	86
RESULT_2 distinct	29
RESULT_3 distinct	34
RESULT_1 x RESULT_2 pairwise intersection	260
RESULT_1 x RESULT_3 pairwise intersection	288
RESULT_2 x RESULT_3 pairwise intersection	257
Proteins common to all samples	248

For questions or comments contact Mark Shifman
Updated 01 Jul 2014 10:28

B

Compare Results: RESULT_1 distinct

RESULT_1: ITRAQ4plex ProGroup DRM_iTRAQ_Set 1_Mar2006 IPI_mouse
RESULT_2: ITRAQ4plex ProGroup DRM_iTRAQ_Set 2_Mar2006 IPI_mouse
RESULT_3: ITRAQ4plex ProGroup DRM_iTRAQ_Set 3_Mar2006 IPI_mouse

86 proteins found, displaying all proteins.
1

Protein ID	Protein Name	RESULT_1 115/114	RESULT_1 116/114	RESULT_1 117/114
IPI00317794	Nucleolin	0.73	1.15	0
IPI00343134	Strawberry notch homolog 2	1.14	1.98	1.94
IPI00755120	similar to Ribosome-binding protein 1	0.72	0.33	0.36
IPI00136723	Adult male testis cDNA, RIKEN full-length enriched library, clone:4933429D13 product:hypothetical protein, [illegible]	0.9	1.01	0.8

C

Compare Results: RESULT_1 x RESULT_2 pairwise intersection

RESULT_1: ITRAQ4plex ProGroup DRM_iTRAQ_Set 1_Mar2006 IPI_mouse
RESULT_2: ITRAQ4plex ProGroup DRM_iTRAQ_Set 2_Mar2006 IPI_mouse
RESULT_3: ITRAQ4plex ProGroup DRM_iTRAQ_Set 3_Mar2006 IPI_mouse

260 proteins found, displaying all proteins.
1

Protein ID	Protein Name	RESULT_1 115/114	RESULT_1 116/114	RESULT_1 117/114	RESULT_2 115/114	RESULT_2 116/114	RESULT_2 117/114
IPI00469621	533 kDa protein	1.05	0.95	1.01	1.15	0.97	1.03
IPI00128689	Collagen alpha-1(V) chain precursor	1.05	0.48	0.49	0.93	0.54	0.5
IPI00380896	Myosin-1	0.85	1.08	1.03	0.86	1.24	1.3
IPI00344004	NADH dehydrogenase (Ubiquinone) 1 alpha subcomplex, 12	1.05	1.17	1.21	1.35	1.49	1.47

D

Compare Results: Proteins common to all samples

RESULT_1: ITRAQ4plex ProGroup DRM_iTRAQ_Set 1_Mar2006 IPI_mouse
RESULT_2: ITRAQ4plex ProGroup DRM_iTRAQ_Set 2_Mar2006 IPI_mouse
RESULT_3: ITRAQ4plex ProGroup DRM_iTRAQ_Set 3_Mar2006 IPI_mouse

248 proteins found, displaying all proteins.
1

Protein ID	Protein Name	RESULT_1 115/114	RESULT_1 116/114	RESULT_1 117/114	RESULT_2 115/114	RESULT_2 116/114	RESULT_2 117/114	RESULT_3 115/114	RESULT_3 116/114	RESULT_3 117/114
IPI00469621	533 kDa protein	1.05	0.95	1.01	1.15	0.97	1.03	1.24	1.09	1.02
IPI00128689	Collagen alpha-1(V) chain precursor	1.05	0.48	0.49	0.93	0.54	0.5	1	0.48	0.6
IPI00380896	Myosin-1	0.85	1.08	1.03	0.86	1.24	1.3	1.52	1.57	1.73
IPI00344004	NADH dehydrogenase (Ubiquinone) 1 alpha subcomplex, 12	1.05	1.17	1.21	1.35	1.49	1.47	1.35	2.14	1.89

Supplemental Figure 5.
