# Supplementary material for: YPED: An Integrated Bioinformatics Suite and Database for Mass Spectrometry-based Proteomics Research
Source: Genomics Proteomics Bioinformatics. 2015 Feb 21;13(1):25–35. doi: 10.1016/j.gpb.2014.11.002 (PMC4411476; doi:10.1016/j.gpb.2014.11.002)
Supplement: Figure S6 — YPED repository A. Search interface. B. Search results for a protein with the ID KCC2G_HUMAN. [file mmc6.pdf]

A

YPED Repository

Menu for YPED Repository Searches

**MS Instrument**	All ▾
**Analysis**	LCMS ▾
**Protein ID**	KCC2G_HUMAN
**Protein Name (partial ok)**	
**Peptide Sequence**	
**Gene Symbols(one per line)**	

Search Protein/Peptide/Gene Symbols

Search Repository Projects help — Search for Projects

Search Repository Results help — Search for Results

To Front Page

For questions or comments contact Mark Shifman
Updated 10 Jun 2014 10:59

B

YPED Repository

Peptide Report LCMS

Seach Term Protein ID KCC2G_HUMAN
The top 3000 peptides are returned

51 peptides identified.

Protein Name	Protein ID	Peptide Score	Expectation	Sequence	Blast Results	Start	End	Delta	ppm	M/Z	Charge	Mascot DAT
Calcium/calmodulin-dependent protein kinase type II subunit gamma OS=Homo sapiens GN=CAMK2G PE=1 SV=3	KCC2G_HUMAN	101	1.4E-8	K.ICDPGLTSFEPEALGNLVEGMDFHK.F	BLAST Results	452	476	-0.0274	-9.9	926.0943	3	download
Calcium/calmodulin-dependent protein kinase type II subunit gamma OS=Homo sapiens GN=CAMK2G PE=1 SV=3	KCC2G_HUMAN	101	1.8E-8	K.NLINQMLTINPAK.R	BLAST Results	247	259	-0.0085	-5.8	735.4066	2	download
Calcium/calmodulin-dependent protein kinase type II subunit gamma OS=Homo sapiens GN=CAMK2G PE=1 SV=3	KCC2G_HUMAN	101	1.8E-8	K.NLINQMLTINPAK.R	BLAST Results	247	259	-0.0085	-5.8	735.4066	2	download
Calcium/calmodulin-dependent protein kinase type II subunit gamma OS=Homo sapiens GN=CAMK2G PE=1 SV=3	KCC2G_HUMAN	101	1.4E-8	K.ICDPGLTSFEPEALGNLVEGMDFHK.F	BLAST Results	452	476	-0.0274	-9.9	926.0943	3	download
Calcium/calmodulin-dependent protein kinase type II subunit gamma OS=Homo sapiens GN=CAMK2G PE=1 SV=3	KCC2G_HUMAN	92	0.0000014	R.LHDSISEEGFHYLVFDLVTGGELFEDIVAR.E	BLAST Results	76	105	-0.0294	-8.6	852.668	4	download
Calcium/calmodulin-dependent protein kinase type II subunit gamma OS=Homo sapiens GN=CAMK2G PE=1 SV=3	KCC2G_HUMAN	83	0.0000012	R.FTDDYQLFEELGK.G	BLAST Results	10	22	1.0036	625.4	803.3794	2	download
Calcium/calmodulin-dependent protein kinase type II subunit gamma OS=Homo sapiens GN=CAMK2G PE=1 SV=3	KCC2G_HUMAN	83	0.0000012	R.FTDDYQLFEELGK.G	BLAST Results	10	22	1.0036	625.4	803.3794	2	download
Calcium/calmodulin-dependent protein kinase type II subunit gamma OS=Homo sapiens GN=CAMK2G PE=1 SV=3	KCC2G_HUMAN	69	0.0000073	K.AGAYDFPSPEWDTVTPEAK.N	BLAST Results	228	246	-0.0009	-0.4	1040.9781	2	download
Calcium/calmodulin-dependent protein kinase type II subunit gamma OS=Homo sapiens GN=CAMK2G	KCC2G_HUMAN	65	0.000024	K.ICDPGLTSFEPEALGNLVEGMDFHK.F + Methyl (C-term); Methyl (K)	BLAST Results	452	476	-0.0279	-10	935.4379	3	download

Supplemental Figure 6.
